# Quality of eyeglass prescriptions from a low-cost wavefront autorefractor evaluated in rural India: results of a 708-participant field study

**DOI:** 10.1136/bmjophth-2018-000225

**Published:** 2019-06-14

**Authors:** Nicholas J Durr, Shivang R Dave, Daryl Lim, Sanil Joseph, Thulasiraj D Ravilla, Eduardo Lage

**Affiliations:** 1 Department of Biomedical Engineering, Johns Hopkins University, Baltimore, Maryland, USA; 2 PlenOptika, Inc, Boston, Massachusetts, USA; 3 Lions Aravind Institute of Community Ophthalmology (LAICO), Aravind Eye Care System, Madurai, India; 4 Department of Electronics and Communications Technology, Universidad Autónoma de Madrid, Madrid, Spain

**Keywords:** diagnostic tests/investigation, optics and refraction, vision, public health

## Abstract

**Objective:**

To assess the quality of eyeglass prescriptions provided by an affordable wavefront autorefractor operated by a minimally trained technician in a low-resource setting.

**Methods and Analysis:**

708 participants were recruited from consecutive patients registered for routine eye examinations at Aravind Eye Hospital in Madurai, India, or an affiliated rural satellite vision centre. Visual acuity (VA) and patient preference were compared between trial lenses set to two eyeglass prescriptions from (1) a novel wavefront autorefractor and (2) subjective refraction by an experienced refractionist.

**Results:**

The mean±SD VA was 0.30±0.37, –0.02±0.14 and −0.04±0.11 logarithm of the minimum angle of resolution units before correction, with autorefractor correction and with subjective refraction correction, respectively (all differences p<0.01). Overall, 25% of participants had no preference, 33% preferred eyeglass prescriptions from autorefraction, and 42% preferred eyeglass prescriptions from subjective refraction (p<0.01). Of the 438 patients 40 years old and younger, 96 had no preference and the remainder had no statistically significant difference in preference for subjective refraction prescriptions (51%) versus autorefractor prescriptions (49%) (p=0.52).

**Conclusion:**

Average VAs from autorefractor-prescribed eyeglasses were one letter worse than those from subjective refraction. More than half of all participants either had no preference or preferred eyeglasses prescribed by the autorefractor. This marginal difference in quality may warrant autorefractor-based prescriptions, given the portable form factor, short measurement time, low cost and minimal training required to use the autorefractor evaluated here.

**Key messages**

**What is already known about this subject?**

- A lack of eye care providers in low-resource settings contributes to a large burden of uncorrected refractive errors.
- Autorefractors are conventionally considered too expensive and inaccurate to significantly to improve refractive eye care capacity in these settings.

**What are the new findings?**

- Eyeglass prescriptions can be accurately measured by a minimally trained technician using a low-cost wavefront autorefractor in rural India.
- Data from 708 participants indicate a marginal difference in both prescription preference and resulting visual acuity between eyeglasses derived from subjective refraction versus autorefraction.
- Among the 438 participants 40 years old and younger, there was no statistically significant difference in the preferences for eyeglasses derived from subjective refraction versus autorefraction.

**How might these results change the focus of research or clinical practice?**

- These results suggest that eyeglasses prescribed objectively by a wavefront autorefractor may be a feasible approach to increasing eyeglass accessibility in low-resource settings.

## Introduction

Over one billion people worldwide suffer from poor vision that could be corrected with a pair of prescription eyeglasses.^1–3^ These uncorrected refractive errors (UREs) are a major cause of lost productivity, limited access to education and reduced quality of life.

The prevalence of UREs is generally highest in low-resource settings, due in part to the severe shortage of eye care professionals.^2 4^ There are several national and international efforts to increase eye care capacities by task-shifting the eyeglass prescription procedure to mid-level personnel called ‘refractionists’.^4–6^ However, these dedicated eye care workers still require several years of training and practice to become proficient,^7^ and it is difficult to retain these skilled workers in poor, rural and remote areas.^8^ There is a need to deskill the refraction process to reduce the training required for refractionist, increase their efficiency and improve the quality of their prescriptions.

Autorefractors are commonly used in high-resource settings to obtain a prescription that is used as a starting point for subjective refraction, reducing the overall time required for a refraction. However, autorefractors are conventionally considered too inaccurate to provide prescriptions without subjective refinement.^9–12^ Previous research comparing patient tolerance and acceptance of eyeglasses has found that approximately twice as many people preferred prescriptions from subjective refraction compared with prescriptions directly from an autorefractor, even after 3 weeks of habituating to the prescribed eyeglasses.^9 10^ A more recent study found a smaller gap in preferences using modern autorefractors on a young adult, non-presbyopic population—in this group, 41% more patients preferred prescriptions from subjective refraction compared with objective methods.^12^ Sophisticated autorefractors based on wavefront aberrometry have been explored for accurate prescriptions, enabled by algorithms incorporating both high-order and low-order aberrations and advanced quality metrics.^13 14^

Despite concerns over accuracy of objective refraction, several groups have developed systems with the goal of augmenting or even substituting for eye care providers in low-resource settings. Some of these approaches include the focometer,^15 16^ adjustable lenses,^15 17^ photorefraction,^18^ inverse Shack-Hartmann systems^19^ and simplified wavefront aberrometers.^20 21^ Previous work has assessed the accuracy of objective autorefractors relative to subjective refraction or conventional commercial autorefractors, but these studies have limited applicability to practical use in low-resource settings because (1) they tested a small population size and age range, (2) participants were highly educated (eg, optometry students), (3) the device was operated by highly trained eye care provider or engineer, (4) the test site was a controlled laboratory without examination time constraints, and/or (5) they excluded patients with comorbidities such as cataracts, keratoconus and conjunctivitis.

We recently introduced an aberrometer that uses low-cost components and calculates a prescription from dynamic wavefront measurements captured from a short video. Measurements from a previous study found that spherical error from this aberrometer agreed within 0.25 dioptres (D) of subjective refraction in 74% of eyes, compared with 49% agreement of the same eyes measured with a Grand Seiko WR-5100K commercial autorefractor.^20^ This prototype is currently under commercial development for low-resource markets (by PlenOptika, USA and Aurolab, India). The goal of this study was to assess the prescription quality from this device under realistic constraints for applicability in low-resource environments. Specifically, we evaluated the performance of this aberrometer when operated by a minimally trained technician in a low-resource setting on a large population of patients registered for routine eye examinations at either a major eye hospital or a satellite vision centre.

## Methods

### Participants

Study objectives and procedures were explained in the local dialect and verbal informed consent was obtained. Written consent was obtained from additional participants for permission to publish photographs depicting them using the autorefractor.

Subjects were recruited from consecutive patients visiting the general ophthalmology unit of Aravind Eye Hospital in Madurai or a rural satellite vision centre in Thiruppuvanam. Inclusion criteria were that patients were between the ages of 15 and 70 years and within the refractive error range of the autorefractor (spherical equivalent of −6 D to +10 D), as determined by subjective refraction. Exclusion criteria included presence of mature cataract, any prior eye surgery, any major eye illnesses, and use of systemic or ocular drugs which may affect vision. The study was completed during the summer of 2015.

### Subjective refraction procedure

Patients who completed a standard-of-care refraction and met the study eligibility criteria were recruited for the study. This included streak retinoscopy and subjective refraction by an experienced refractionist. Refractions at the Aravind base hospital also included measurements by a standard commercial autorefractor before the subjective refraction. Subjective refraction was performed using a trial lens set and a digital visual acuity (VA) chart (Aurolab Aurochart) placed 3 m away from the participant.

### Autorefractor procedure

A technician with experience in coordinating eye research studies but no training in refraction or clinical optometry was trained to use the prototype autorefractor in two 2-hour sessions, followed by 4 hours of practice refractions with the goal of consistently administering verbal instructions to the participants. All participants were tested by this technician. The autorefractor was calibrated at the beginning of the study. No recalibration was performed throughout the 3-month study duration, which included daily packing, unpacking and transportation. Every autorefractor measurement was performed directly after standard-of-care subjective refraction at a second station in a different room.

**Figure 1:**
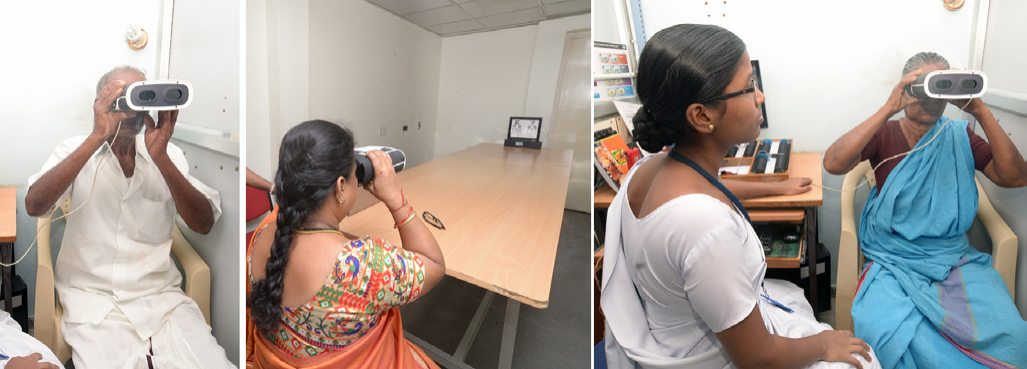
Testing procedure for the wavefront autorefractor. Participants looked through the open-view wavefront autorefractor at a distant back-lit visual acuity chart, while three 10 s videos of wavefront images were recorded by the device. The autorefractor was flipped over to measure the opposite eye. After repeating three times, the system displayed the autorefractor eyeglass prescription.

Participants were instructed to hold the autorefractor to their face, rest their elbows on a table for support and look through the device at a back-lit VA chart placed 3 m away (figure 1). The technician adjusted the interpupillary distance wheel on the autorefractor and manually adjusted the pitch of the device until the participant could see a red spot coming from the autorefractor. When the participant saw a bright red spot within, the technician turned on the VA chart and began recording a 10 s video of wavefront measurements with the autorefractor. The participant was instructed to blink whenever desired and to look at the VA chart during the video. After the video was acquired, the device was flipped upside down to measure the opposite eye and the procedure was repeated. The participant was then measured two additional times for a total of three measurements of each eye. After the first interpupillary distance adjustment was made, typically no further adjustments were necessary. The device computed the median of the three measurements and displayed this prescription in the same format as subjective refraction on a companion laptop.

### Prescription quality assessment

Sphere, cylinder and axis values were transcribed from the subjective refraction and autorefractor measurements to an electronic database, which randomly assigned them to prescriptions ‘A’ or ‘B’. The participant was escorted to a third station for VA measurement and preference survey by an experienced refractionist that was not involved in either prior refraction. This refractionist measured the VA of each eye using trial lenses set to each prescription pair in a randomised sequence, using a digital VA chart placed 3 m from the participant. The refractionist then asked the participant which prescription they preferred: A, B or no preference. VA and preference results were entered into an electronic database that used a de-identified numeric code to track each participant.

### Statistical analysis

For statistical comparison, prescriptions were converted to power vector parameters of spherical equivalent (*M*), vertical Jackson cross cylinder (*J*_*0*_) and oblique Jackson cross cylinder (*J*_*45*_) for subjective refraction (*M*_*SR*_, *J*_*0,SR*_, *J*_*45,SR*_) and autorefraction (*M*_*AR*_, *J*_*0,AR*_, *J*_*45,AR*_). Given that subjective refraction has significant interoptometrist and intraoptometrist variation,^22^ we performed a Bland-Altman analysis to assess correlation, bias and outliers between the two measurements for each power vector component. We computed the 95% limit of agreement between the two measurements using the approximation of the average difference ± (1.96 × SD) of the differences. We tested the effect of age in the disagreement between subjective refraction and autorefraction by fitting a linear model with slope, m, and intercept, b, to the absolute difference of power vector measurements of the right eye of each patient, and assessed the influence of gender and testing site as covariates. We also compared the average anisometropia measured by subjective refraction and autorefraction by comparing the difference in power vectors measured in the right and left eye of each patient.

All VA measurements were converted to logarithm of the minimum angle of resolution (logMAR) units for statistical comparison. VAs from uncorrected vision (*VA*_*UC*_), correction by autorefractor-determined prescription (*VA*_*AR*_) and correction by subjective refraction-determined prescription (*VA*_*SR*_) were compared using a box and whisker plot of results from the right eyes only to avoid the influence of isometropia on the independence of the samples. Differences between mean values were assessed with a Wilcoxon signed-rank test with a significance level of 0.05. The participant survey for prescription preference was evaluated using a z test of proportion with a significance level of 0.05. Both VA and prescription preference results were analysed for the entire population and within two age groups partitioned by the estimated age of onset of presbyopia of 40 years of age.^23^

## Results

### Participants

We enrolled 506 participants from the base hospital and 202 participants from the vision centre. All 708 participants successfully received a testable prescription from both the prototype autorefractor and the subjective refraction. Within our study population, 220 participants had presbyopia, 89 participants had at least one immature cataract, 21 participants had conjunctivitis, and 1 participant had keratoconus. The mean±SD age of participants was 35±13 years, 438 participants were 15–40 years of age, 270 participants were 41–70 years of age, and 413 participants were female. A summary of the patient population, including a description of the distribution of refractive errors by gender, is presented in table 1.

**Table 1:** Participant demographics and average refractive error measurements

Gender	n (%)	Age±SD (range) (years)	Subjective refraction			Objective refraction		
			*M* (D) (5%–95%)	*J*_*0*_(D) (5%–95%)	*J*_*45*_(D) (5%–95%)	*M* (D) (5%–95%)	*J*_*0*_(D) (5%–95%)	*J*_*45*_(D) (5%–95%)
Female	413 (58)	34±12 (15–65)	−0.48 (−3.75 to 1.50)	−0.02 (−0.50 to 0.42)	0.00 (−0.13 to 0.16)	−0.55 (−4.36 to 1.50)	0.00 (−0.38 to 0.49)	0.04 (0.00–0.13)
Male	296 (42)	37±14 (15–70)	−0.59 (−3.50 to 1.00)	−0.03 (−0.50 to 0.38)	0.00 (−0.16 to 0.22)	−0.71 (−4.38 to 1.25)	−0.03 (−0.49 to 0.37)	0.04 (0.00–0.15)

5%, 5th percentile; 95%, 95th percentile; D, dioptres; *J_0_*, vertical Jackson cross cylinder; *J_45_*, oblique Jackson cross cylinder; *M*, spherical equivalent.

### Patient and public involvement

Patient participants were not involved in defining the research questions, developing the outcome measures, designing the study or in the recruitment. We conducted two pilot studies involving real patients, and their responses and feedback were used to modify the data collection protocol. The results of the study will be made available to the participants in the form of summary (written in lay language).

### Prescription agreement

**Figure 2:**
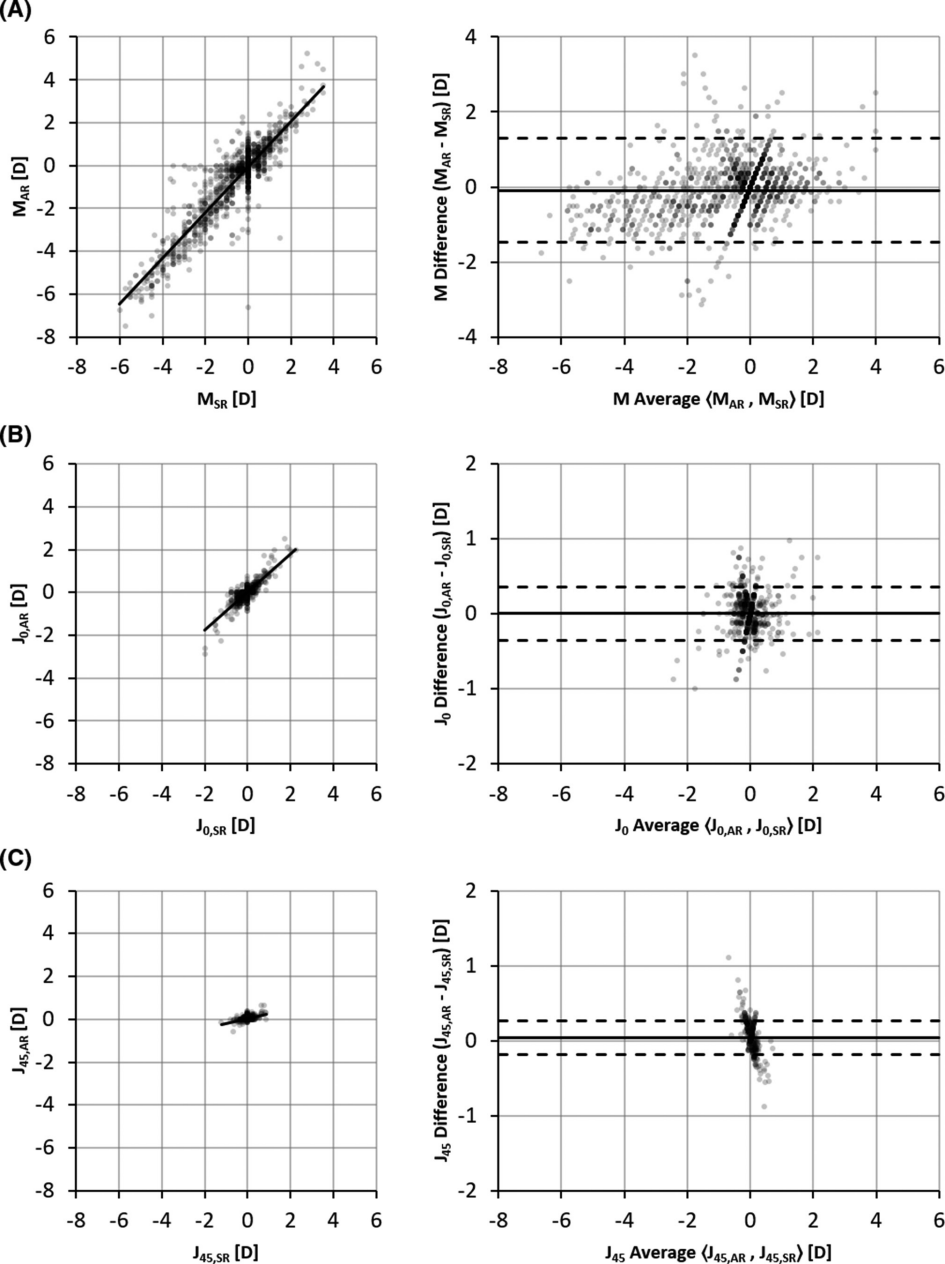
Correlation and Bland-Altman plots of power vectors measured by autorefractor versus subjective refraction. Correlation (left) and Bland-Altman (right) plots comparing agreement of prescriptions measured by subjective refraction and the prototype autorefractor for (A) spherical equivalent, *M*, (B) vertical Jackson cross cylinder, *J*_*0*_, and (C) oblique Jackson cross cylinder, *J*_*45*_. AR, autorefractor prescription; SR, subjective refraction prescription; D, dioptre.

We observed a strong correlation between prescriptions from subjective refraction and the autorefractor, with Pearson linear correlation coefficients of r=0.94, r=0.83 and r=0.40 for *M*, *J*_*0*_ and *J*_*45*_, respectively (r^2^ values of 0.83, 0.70 and 0.16) (figure 2). The smaller correlation coefficient for *J*_*45*_ was likely influenced by the small range of values in the study population. The SD of *J*_*45*_, measured by subjective refraction, was only 0.12 D, compared with 1.46 D and 0.30 D for *M* and *J*_*0*_, respectively. In the correlation plot for figure 2A, one measurement (−3.75, –8.25) falls outside of the viewable range.

From Bland-Altman analysis, we observed a bias between the subjective refraction and autorefractor measurements of −0.09 D, 0.01 D and 0.04 D for *M*, *J*_*0*_ and *J*_*45*_, respectively (figure 2), with the autorefractor reporting more myopic spherical equivalent values on average than subjective refraction. There was also a trend for larger magnitude measurements of both myopia and hyperopia by the autorefractor. A linear fit to the Bland-Altman data has a slope of 0.16 and an R of 0.36 (line not shown), signalling either a general undercorrection from subjective refraction or an overestimation of refractive error power measurement by the autorefractor. The 95% limits of agreement between the two methods were −1.47 D to 1.30 D, −0.35 D to 0.36 D, and −0.19 D to 0.27 D for *M*, *J*_*0*_ and *J*_*45*_, respectively. In the Bland-Altman plot for figure 2A, three measurements ((−6.00, –4.50), (−3.31, –6.63) and (−0.94, –4.88)) fall outside of the viewable range. Analysing the absolute difference of the subjective refraction versus autorefraction power vector measurements, we observed weak relationships with age, gender and site of measurement (table 2). The slope of linear fits to these data was in all cases less than or equal to 0.003 D per year of age, indicating a maximum change in average discrepancy between the two measurement techniques of 0.09 D in patient populations separated by a 30-year age difference, which is smaller than the 0.25 D rounding increments of the two approaches. For each power vector component, the average discrepancy between refractive approaches was within 0.08 D when comparing male versus female participants, and within 0.04 D when comparing refraction at Aravind Eye Hospital versus the satellite vision centre. The average difference between refraction of the right and left eye of each patient was 0.02 D, 0.00 D and 0.00 D for *M*, *J*_*0*_ and *J*_*45*_, respectively, for both subjective refraction and autorefraction, indicating no systematic difference in measurements between the two eyes for the two approaches.

**Table 2:** Trends in absolute difference of subjective refraction versus autorefraction of right eye measurements

	All right eyes			Female			Male			Hospital			Vision centre		
	Avg (D)	m (D/year)	b (D)	Avg (D)	m (D/year)	b (D)	Avg (D)	m (D/year)	b (D)	Avg (D)	m (D/year)	b (D)	Avg (D)	m (D/year)	b (D)
|*M*_*SR*_ -*M*_*AR*_|	0.482	0.001	0.43	0.448	0.001	0.43	0.529	0.002	0.47	0.492	0.001	0.46	0.455	0.002	0.39
|*J*_*0*,*SR*_-*J*_*0*,*AR*_|	0.110	0.002	0.04	0.111	0.003	0.02	0.109	0.001	0.06	0.116	0.001	0.07	0.096	0.004	−0.03
|*J*_*45*,*SR*_-*J*_*45*,*SR*_|	0.063	0.001	0.05	0.061	0.001	0.03	0.067	0.000	0.06	0.077	0.000	0.07	0.028	0.000	0.02

AR, autorefractor prescription; Avg, average; D, dioptre; *J_0_*, vertical Jackson cross cylinder; *J_45_*, oblique Jackson cross cylinder; *M*, spherical equivalent; SR, subjective refraction prescription; b, intercept of linear fit; m, slope of linear fit.

### Visual acuity

**Figure 3:**
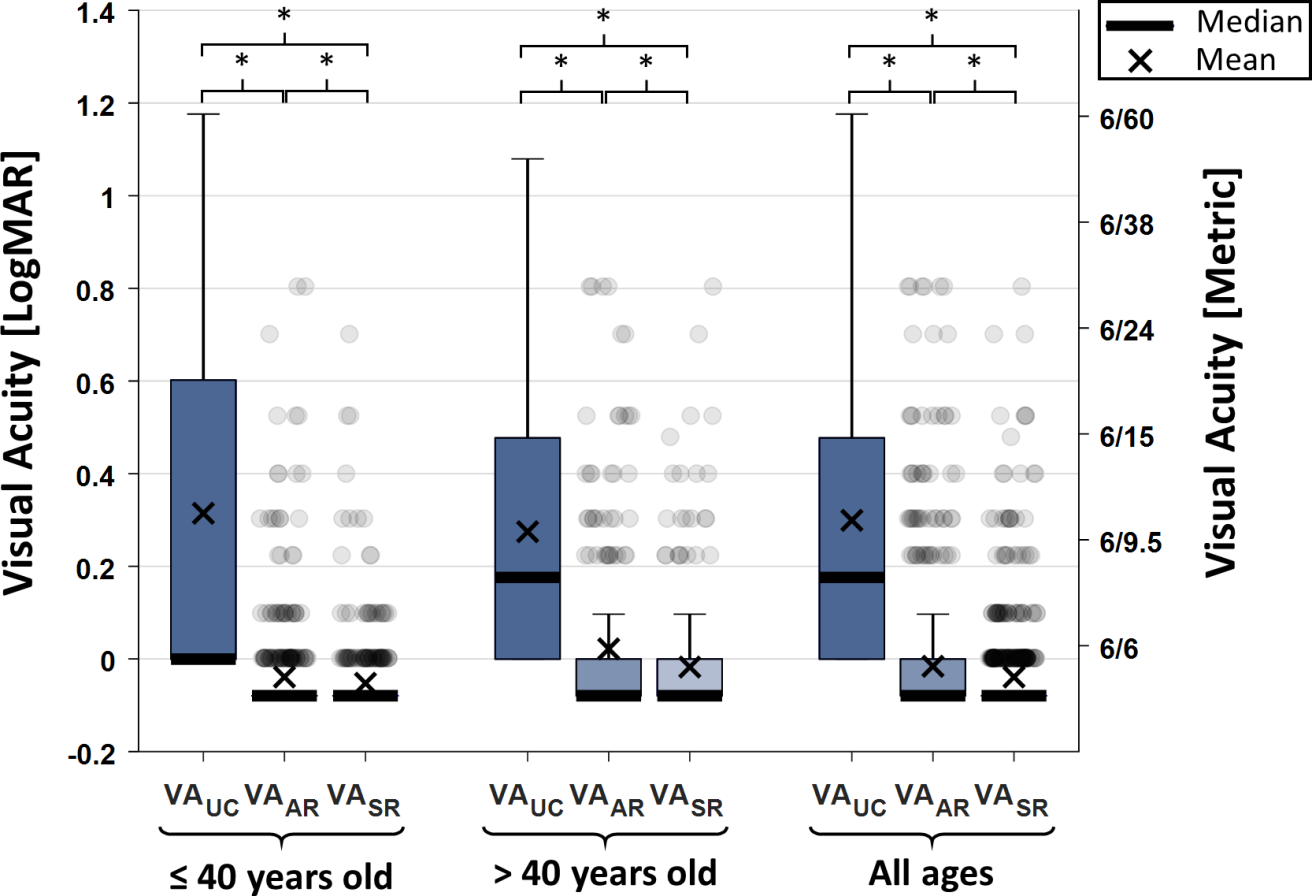
Box plot of visual acuity before and after correction. Visual acuity of the right eyes without correction (*VA*_*UC*_), with trial lenses set to the autorefractor-determined prescription (*VA*_*AR*_) and with trial lenses set to the subjective refraction-determined prescription (*VA*_*SR*_). There was a statistically significant difference (p<0.01) between average visual acuity measurements among all combinations within each age group (indicated by *). logMAR, logarithm of the minimum angle of resolution.

We measured a mean±SD of 0.30±0.37, –0.02±0.14 and −0.04±0.11 logMAR units for *VA*_*UC*_, *VA*_*AR*_ and *VA*_*SR*_, respectively. VA distributions for the whole study population as well as the age-grouped populations are shown in figure 3. VA was better after correction from both refraction methods (p<0.01) for all study groups. *VA*_*SR*_ was also better than *VA*_*AR*_ (p<0.01) for all study groups, by margins of 0.01, 0.04 and 0.02 logMAR units for the younger, older and all age groups, respectively.

### Prescription preference

Overall, 25% of participants had no preference of eyeglasses, 42% preferred prescriptions from subjective refraction, and 33% preferred prescriptions from the autorefractor (table 3). The entire population and the older groups preferred subjective refraction prescriptions more often than autorefractor prescriptions (p<0.01). Within the 342 participants in the younger group that had a preference, there was no statistically significant difference in prescription preference (49% preferred autorefractor prescriptions, 51% preferred subjective refraction prescriptions; p=0.52).

**Table 3:** Participant preference of trial lens prescriptions with masked origin

Age group	Participants, n (%)				P value SR vs AR preference
	All	No preference	Preferred SR	Preferred AR	
15–40	438 (61.9)	96 (21.9)	174 (39.7)	168 (38.4)	0.52
41–70	270 (38.1)	82 (30.4)	123 (45.6)	65 (24.1)	<0.01
All	708 (100.0)	178 (25.1)	297 (41.9)	233 (32.9)	<0.01

AR, autorefractor prescription; SR, subjective refraction prescription.

## Discussion

This study found smaller differences in VA and preference of prescriptions obtained from autorefraction compared with subjective refraction than previous work.^9–12^ There are several differences to our study design and autorefractor that may contribute to this result. The refractionists used in our study specialise in high-volume refractive eye exams and have less training than optometrists or ophthalmologists used in other studies. Our study used a 3 m refraction distance since it is the standard of care within the Aravind system, but the convention of most eye exams is a 6 m or 20-foot distance. Our study was also conducted on an Indian population in a low-resource setting, which could have systematic differences in VA preferences and compliance to subjective refraction instructions. The autorefractor tested in our study is significantly different from previous studies. It is an open-view wavefront aberrometer that analyses wavefront data from three 10 s videos of measurements (typically 240 wavefronts), rather than a single snapshot or the average of several images. Lastly, this autorefractor prototype had a spherical equivalent refractive error range of −6 D to +10 D, and only patients within this range were recruited for this study. Therefore, the conclusion of these results is relevant only to patients within this range tested.

This study is, to the best of our knowledge, the first that identifies a population (patients 40 years old and younger) that exhibits no statistically significant difference between preferences of prescriptions derived from an autorefractor compared with subjective refraction. The difference in preference between the two age groups may be due to several physiological parameters that vary with age. While patients with mature cataracts were excluded from this study, 6 patients (1.4%) in the younger group were noted to have at least one immature cataract, while 83 patients (30.7%) in the older group were noted to have at least one immature cataract. Pupil size was not directly measured here, but is known to decrease significantly with age.^24^ Both opacities in the lens and a small pupil make the projection of the wavefront beacon on the retina and the measurement of the emerging wavefront more difficult. The older group is also expected to have smaller accommodative amplitude. Closed-view wavefront autorefractors are known to cause instrument-induced myopia, leading to an overestimation of myopia.^25^ However, the system evaluated here is open-view and the observed trend was of greater autorefractor prescription preference in the population expected to have larger accommodation amplitude. Lastly, the technological literacy and compliance to both the subjective refraction and autorefraction procedures may differ between the age groups, both of which could influence the quality of the prescriptions from each method. We observed that participant age, gender and site were weakly related to the difference between refractive errors measured by subjective refraction and autorefraction, indicating that the increasing preference for subjective refraction-determined prescriptions with older age is likely of multifactorial origin and cannot be explained by simple systematic differences in the prescriptions from the two approaches.

In this study, we only surveyed participants for nominal prescription preference. Future work assessing the qualitative strength of preference and satisfaction of each prescription with ordinal surveys is under way and will provide more insight into differences in perceived quality of the prescriptions. We also assessed VA and preference immediately after the eye examination with trial lenses, but assessing prescription quality after several weeks of habituation to the prescription eyeglasses will improve the understanding of factors influencing long-term patient satisfaction. Lastly, a new version of the prototype autorefractor evaluated in this study is currently being commercialised with a larger refractive range, improved ergonomics and is targeted to be cost-effective for low-resource settings.

Participants using eyeglasses prescribed by the autorefractor operated by a non-clinical, minimally trained technician achieved a VA that was only approximately one letter worse than using eyeglasses prescribed by an experienced refractionist. Moreover, although participants preferred subjective refraction prescriptions in aggregate, participants 40 years of age and younger had no statistically significant difference in their preference. Given the minimal training required to use the autorefractor tested here and the marginal difference in prescription quality by the refractionist compared with the autorefractor, wavefront-based objective prescriptions may be a viable substitute for subjective refraction in low-resource settings.
